# The Role of Chemokines in Wound Healing

**DOI:** 10.3390/ijms19103217

**Published:** 2018-10-18

**Authors:** Anisyah Ridiandries, Joanne T. M. Tan, Christina A. Bursill

**Affiliations:** 1 Department of Cardiology, Kolling Institute, Northern Sydney Local Health District, St Leonards, NSW 2065, Australia; anisyah.ridiandries@sydney.edu.au; 2 Sydney Medical School Northern, University of Sydney, Sydney, NSW 2006, Australia; 3 Heart Health Theme, South Australian Health and Medical Research Institute, North Terrace, Adelaide, SA 5000, Australia; Joanne.Tan@sahmri.com; 4 Adelaide Medical School, Faculty of Health & Medical Sciences, University of Adelaide, Adelaide, SA 5000, Australia

**Keywords:** chemokine, wound, healing, angiogenesis, inflammation

## Abstract

Wound healing is a multistep process with four overlapping but distinct stages: hemostasis, inflammation, proliferation, and remodeling. An alteration at any stage may lead to the development of chronic non-healing wounds or excessive scar formation. Impaired wound healing presents a significant health and economic burden to millions of individuals worldwide, with diabetes mellitus and aging being major risk factors. Ongoing understanding of the mechanisms that underly wound healing is required for the development of new and improved therapies that increase repair. Chemokines are key regulators of the wound healing process. They are involved in the promotion and inhibition of angiogenesis and the recruitment of inflammatory cells, which release growth factors and cytokines to facilitate the wound healing process. Preclinical research studies in mice show that the administration of CCL2, CCL21, CXCL12, and a CXCR4 antagonist as well as broad-spectrum inhibition of the CC-chemokine class improve the wound healing process. The focus of this review is to highlight the contributions of chemokines during each stage of wound healing and to discuss the related molecular pathologies in complex and chronic non-healing wounds. We explore the therapeutic potential of targeting chemokines as a novel approach to overcome the debilitating effects of impaired wound healing.

## 1. Introduction

Wound healing is a multistep process requiring the strict coordination of multiple cell types and molecular signaling molecules at the wound site. This process can be divided into four distinct but overlapping stages: hemostasis, inflammation, proliferation, and remodeling. Hemostasis is the first stage of wound healing, in which a clot forms to prevent further blood loss. The next stage of healing is inflammation, in which neutrophils and macrophages are recruited to remove debris from the wound site to prevent infection. Following this is the proliferation stage, where re-epithelialization and granulation occur by recruitment of several cells including stem cells, endothelial cells, and keratinocytes to close the wound. Angiogenesis, the formation of new blood vessels from pre-existing vessels, is important during both the inflammation and proliferation phases of wound healing. Whilst nearby post-capillary venules play the largest role, wound neovascularization post-wounding assists in the recruitment of inflammatory cells to the wound for debris removal, causes invasion of the fibrin/fibronectin-rich wound clot, and reorganizes a new microvascular network to maintain the granulation tissue being formed [1]. The final stage in the wound healing process is remodeling, when fibroblasts reorganize the collagen matrix forming a sturdy wound seal. Once the wound closure is complete, the remodeling process continues over several months. Scar tissue is created during this process. In order for the wound to close efficiently, these events must occur in sequence. Chemokines play a key role in orchestrating this sequence through the regulation of angiogenesis and the recruitment of inflammatory cells which secrete cytokines and growth factors to promote wound healing.

Chemokines are small (8–12 kDa) chemotactic cytokines that regulate the migration of cells to the site of injury. Chemokines are secreted by a wide variety of cells in the wound, including endothelial cells, fibroblasts, keratinocytes, neutrophils, and macrophages. They are divided into four groups based on the placement and number of cysteine residues at the N-terminal: the CC-chemokine group has two cysteine residues adjacent to each other, and the CXC-chemokine group has two cysteine residues separated by an amino acid [2]. This group can be further separated based on the presence of glutamic acid-leucine-arginine (ELR) motif, into ELR− (angiostatic) and ELR+ (angiogenic) [2]. The CX_3_C-chemokine group has three amino acids between two cysteine residues and the C-chemokine group has only one cysteine residue at the N terminal. Of the four chemokine groups, the largest group is the CC-chemokine class (28 members), followed by the CXC-chemokine class (17 members), with the CX_3_C- and C-chemokine classes having 1 and 2 members, respectively.

In wound healing, chemokines are primarily involved in orchestrating the prevention or promotion of angiogenesis at each different healing stage. Angiogenesis is prevented at the hemostasis stage to allow for clot formation, while angiogenesis is promoted in the inflammatory phase to promote the migration of inflammatory cells in and out of the wound. During the proliferation phase, neovessels are needed to meet the metabolic requirements of the proliferating cells during re-epithelialization and granulation. However, in the remodeling phase, neovessel regression is more important to allow for the reorganization of the collagen matrix to form scar tissue. This changing state of angiogenesis requires precise coordination of cytokines, chemokines, and growth factors, making chemokines extremely important in wound healing.

An imbalance in the chemokine environment may alter the wound healing process to cause either prolonged healing or excessive scar formation. Prolonged healing may occur in diseases such as diabetes, where there is an excess of inflammation preventing the wound from progressing to the proliferation stage, leading to the development of a chronic wound that either does not heal or heals with a remaining scar. Excessive scarring, such as with hypertrophic scars, occurs when there is an overproduction of collagen in the wound. In this case, the scar often becomes thick, raised, and may be red in color.

Given the importance of chemokines to the wound healing process, there is therapeutic potential to target chemokines as a treatment for faster healing with reduced scar formation. Several studies have explored the treatment of wounds by either inhibiting or promoting individual chemokines.

## 2. The Roles of Chemokines at Each Stage of the Wound Healing Process

**Figure 1:**
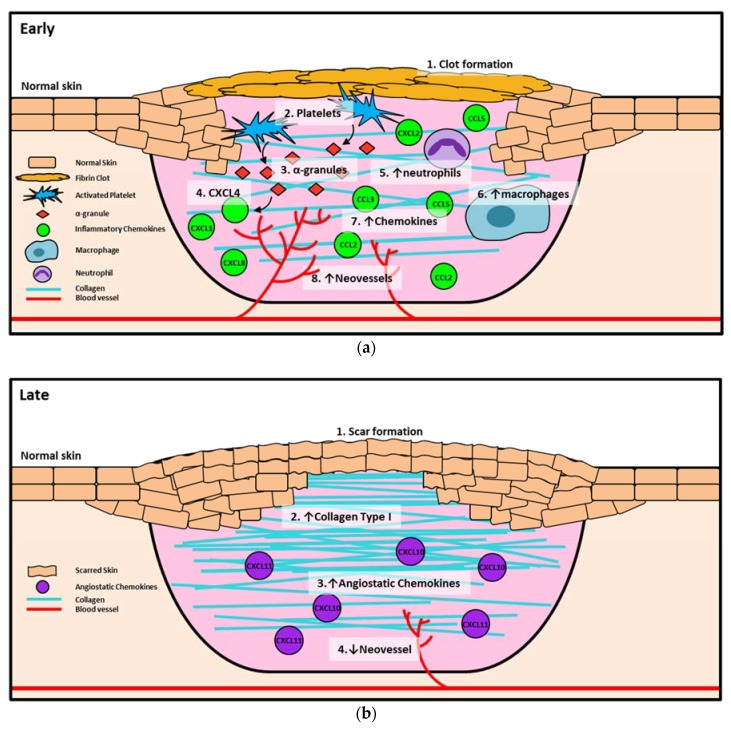
Chemokines in early and late phases of wound healing. (**a**) Early wound healing, including clot formation, inflammation, and proliferation. (1) Clot formation occurs to prevent the loss of blood and (2) platelets are activated and release (3) α-granules, which in turn release (4) CXCL4 as an early inhibitor of angiogenesis. Once the clot has fully formed other, chemokines such as CXCL8, CXCL1, and CXCL2 are released by α-granules to recruit inflammatory cells, including (5) neutrophils and (6) macrophages. Neutrophils are increased early in the healing process, then macrophages soon take over as the primary inflammatory cell. Neutrophils and macrophages release (7) chemokines such as CCL2, CCL3, and CCL5 into the wound to promote the recruitment of more inflammatory cells that release pro-angiogenic growth factors that (8) increase neovessel formation in the wound. (**b**) Late wound healing is the remodeling stage. In this stage, the wound is fully healed and (1) a scar has formed. Type 3 collagen converts to (2) type 1 collagen to promote scar formation and create a more stable wound seal. During the remodeling process (3), angiostatic chemokines (CXCL10, CXCL11) promote the (4) regression of neovessels, as there is no longer a requirement for enhanced blood flow or the recruitment of immunological cells to the site. ↓: indicates decrease;↑: indicates decrease.

Chemokines are involved in all stages of wound healing but are most abundant and most varied during the inflammation and proliferation stages to promote angiogenesis (Figure 1). They are, however, also present in the hemostasis and remodeling phase and work to inhibit the angiogenic process (Table 1).

### 2.1. Chemokines in Hemostasis Stage of Wound Healing

The hemostasis stage is an essential step that prevents further blood loss from the wound site. This involves immediate activation of the coagulation cascade to allow for clot formation. During platelet activation, CXCL4 (also known as Platelet factor 4, PF4) is released from α-granules to inhibit angiogenesis. Other than being angiostatic [10,11], CXCL4 has been shown to inhibit hematopoiesis [16] and collagenase activity [17]. CXCL4 inhibits angiogenesis through binding to vascular endothelial growth factor (VEGF) and basic fibroblast growth factor (bFGF, also known as FGF-2), both potent promoters of angiogenesis, and through binding to its receptor CXCR3. When CXCL4 binds to VEGF and bFGF, it prevents binding to its receptors by disrupting cell surface heparin sulfates and is found to inhibit VEGF-induced proliferation of vascular endothelial cells [18,19]. Tubule formation, induced by VEGF, is found to be inhibited in the presence of CXCL4 in dermal human microvascular endothelial cells and in a human microvascular endothelial cell line [20]. Similarly, the CXCL4 variant, CXCL4L1, inhibited tubule formation in the presence of VEGF and bFGF [21]. CXCL4L1 was also more effective than CXCL4 in inhibiting bFGF-induced angiogenesis in rat corneas [22], and both forms inhibit bFGF-induced bovine aortic endothelial cell (BAOEC) proliferation and human umbilical vein endothelial cell (HUVEC) motility, with CXCL4L1 found to be 20 times more potent than CXCL4 [23]. Additionally, through binding to the receptor CXCR3, the CXCL4L1 variant was found to disrupt targeted cell migration towards CCL5 when compared to CXCL4 [21]. Interestingly, CCL5 and CXCL4 have been shown to have a heterophilic interaction that blocks CCL5-directed chemotaxis of monocytes on the endothelium [24]. Furthermore, CXCL4 and CXCL4L1 were found to be poorly chemotactic to T cells and monocytic THP-1 cells [23]. Binding of CXCL4 to the CXCR3-B receptor variant was shown to upregulate human microvascular endothelial cell (HMEC-1) apoptosis [25] by activation of p38 and μ-calpain cleavage of integrins [20,26]. This is another endothelial cell function that causes inhibition of wound angiogenesis early post-wounding.

Whilst CXCL4 is the predominant chemokine released by platelet α-granules, CXCL1, CXCL4, CXCL5, CXCL7, CXCL8, CXCL12, CCL2, CCL3, and CCL5 are also released to a lesser extent [3]. This becomes important once the fibrin clot has formed, allowing the recruitment of inflammatory cells to initiate the next phase of wound healing.

### 2.2. Chemokines in the Inflammatory Stage of Wound Healing

The inflammatory phase is characterized by an influx of inflammatory cells and an increase of pro-angiogenic molecules in the wound. The main objective of chemokines in this phase is to recruit these inflammatory cells to remove dead cells, debris, and foreign bodies from the wound and to promote the release of pro-angiogenic molecules to facilitate the migration, proliferation, and differentiation of endothelial cells, endothelial progenitor cells (EPCs), and keratinocytes which eventually close the wound.

The initial wave of inflammatory cells is recruited by CXCL8, CXCL1, and CXCL2 that are released by platelet α-granules [3,5,12]. CXCL8 is a potent neutrophil attractant, with neutrophils constituting nearly 50% of cells in the early stages of the wound [4]. In aged rats, the influx of neutrophils was dramatically reduced compared to young rats in the first 4 days post-wounding, causing delayed healing in older rats, indicating the importance of their early recruitment [27]. Neutrophils begin the phagocytosis of debris in the wound and release chemokines including CCL2, CCL3, and CCL5, which recruit macrophages to the wound [5]. Similarly, endothelial cells and keratinocytes already present in the wound border release chemokines to recruit macrophages to the wound site to further promote angiogenesis in the wound [28,29]. Macrophages quickly take over this process, targeting the dying cells, apoptotic neutrophils, and foreign bodies. The large influx of neutrophils and macrophages occurs quickly to prevent the risk of wound infection.

After day 2–4 post-wounding, neutrophils are markedly reduced in the wound, leaving macrophages as the dominant inflammatory cells in the wound, persisting for approximately 14 days post-wounding [4]. Macrophages are important in the wound not only to keep the wound free from invading microorganisms but also to promote the repair of the wound. Macrophages release growth factors, cytokines, and chemokines such as VEGF, bFGF, platelet derived growth factor (PDGF), tumor necrosis factor-α (TNF-α), and interferon-γ (IFN-γ) that stimulate angiogenesis [30,31,32]. Additionally, macrophages release chemokines such as CCL2 and CCL5 to promote the migration of more macrophages to the wound.

Chemokines found in the inflammatory phase are responsible for the recruitment of macrophages and the promotion of angiogenesis. CC-chemokines found in the first week after the initial wounding event include CCL1, CCL2, CCL3, CCL4, CCL5, and CCL7, which are all able to chemoattract macrophages, thereby suggesting a high content of macrophages in the wound [4,33]. CXC chemokines are also present in the wound, including CXCL1, CXCL2, CXCL5, CXCL7, CXCL8, and CXCL12, and are known to directly promote angiogenesis [13]. CXCL1, CXCL2, CXCL5, and CXCL12 are also released by macrophages. Angiogenesis is essential in this phase to facilitate the migration of cells into the wound and to meet the metabolic needs of the proliferating wound cells.

In addition to the induction of inflammation by chemokines, other molecules known as damage associated molecular patterns (DAMPs), which signal cell distress, have also been shown to promote the inflammatory response following wounding [34]. One such DAMP is HMGB1, which is active in late inflammation or in response to stimulation by LPS, TNF-α, or IL-1β [35,36,37]. HMGB1 is secreted by monocytes and macrophages [38], increases the expression of CXCL8 and CCL2 [38], and is involved in the recruitment of EPCs [39]. In response to wounding, HMGB1 expression is increased in normal mice, but in diabetic mice with impaired healing, HMGB1 is decreased [40]. Additionally, HMGB1 treatment enhanced wound healing in diabetic mice by increasing re-epithelialization and increasing the formation of granulation tissue [40]. In vitro, HMGB1 enhanced the migration of keratinocytes and fibroblasts, which is important for tissue repair [40]. Recently, HMGB1 has been found to create a heterocomplex with CXCL12 [41]. When in the heterocomplex formation, HMGB1 is able to enhance the ability of CXCL12 to induce monocyte migration in vitro through the CXCR4 chemokines receptor [41]. Given the role of HMGB1 in several key steps of the inflammatory stage in wound healing, further research is necessary to fully elucidate the role of the HMGB1-CXCL12 complex as well as other DAMP-chemokine complexes in wound healing.

As healing progresses, macrophages switch phenotypes from an M1 pro-inflammatory phenotype to an M2 pro-repair phenotype [42]. This switch in phenotype causes the reduction of inflammatory markers, including TNF-α and NF-κB, and promotes the production of TGF-β, VEGF, and bFGF to further increase angiogenesis and promote cellular proliferation and migration that support changes in the extracellular matrix (ECM) in the proliferation phase.

### 2.3. Chemokines in the Proliferation Stage of Wound Healing

The third phase of wound healing occurs within the 3–10-day period after the initial wounding event. This phase is characterized by increased numbers of endothelial cells, keratinocytes, fibroblasts, and collagen in the wound area, which contribute to the formation of a temporary extracellular matrix (ECM). There is also a reduction in the number of inflammatory cells to promote the re-epithelialization process that closes the wound. In the first few days of the proliferation phase, many neovessels are present to support the rapid increase in cellular proliferation and migration in the wound. This is mediated through pro-angiogenic chemokines CXCL1, CXCL2, CXCL3, CXCL5, CXCL6, CXCL7, and CXCL8 and their receptors CXCR1 and CXCR2 [9]. High expression levels of CXCL1 and CXCL8 have been associated with early wound neoangiogenesis in human wounds [4]. Additionally, CXCR2 knockout mice exhibit reduced neutrophil recruitment, reduced keratinocyte migration, reduced proliferation, and reduced neovascularization during re-epithelialization [43]. Chemokines also play an indirect role in the proliferation stage, facilitating the recruitment of macrophages that secrete growth factors to promote angiogenesis. Chemokines CCL2 and CCL3 are highly expressed in wounds at this stage and coincide with an increased presence of macrophages [4,6,7].

During the re-epithelialization phase, basal keratinocytes express CXCL10 and CXCL11, suggesting an important role of these chemokines [44]. This is highlighted in CXCR3 knockout mice, the receptor for CXCL10 and CXCL11, in which wound re-epithelization and basement membrane regeneration is delayed in both partial and full thickness wounds [44]. These mice also had reduced epidermal maturation compared to wild-type wounds. Conversely, keratinocyte migration, wound closure, and granulation tissue were significantly increased in the presence of fibrin functionalized with fibronectin and CXCL11 in 10-day full thickness mouse wounds [45]. Additionally, CCL27 has been reported to promote the migration of bone-marrow-derived keratinocyte stem cells in full thickness wounds, resulting in faster wound healing [46].

CXCL12 promotes the recruitment of bone-marrow-derived stem cells which differentiate into endothelial cells and fibroblasts to form the granulation tissue [14,15]. The granulation tissue fills the wound from the base up to form a new ECM ready for the deposition of collagen. While this occurs, keratinocytes and endothelial cells at the wound edge proliferate and migrate to close the wound surface. This is stimulated by the release of TGF-β from M2 macrophages. Additionally, TGF-β increases endothelial, fibroblast, and keratinocyte cell proliferation and migration [47,48,49,50,51]. TGF-β is also important for collagen formation, remodeling of the extracellular matrix, and the initiation of granulation tissue formation that stimulates the contraction of fibroblasts, which is important for wound closure [51,52,53,54].

### 2.4. Chemokines in the Remodeling Stage of Wound Healing

The remodeling phase is the longest phase occurring for several months or years after wounding. The processes of angiogenesis and proliferation cease, excess cells either leave the wound or undergo apoptosis, and neovessels undergo regression, leaving mostly collagen and ECM proteins in the wound. During this process, the ECM is broken down by matrix metalloproteinases (MMPs) and metalloproteinase tissue inhibitors (TIMPs), allowing for neovessel regression and the deposition of type I collagen [55]. The type III collagen deposited during the proliferation phase is degraded and replaced with stronger, thicker, more permanent type I collagen forming the final scar [56]. In vitro CCL2 was found to promote expression of MMP-1 and TIMP-1 in human dermal fibroblasts, indicating a profibrotic and collagen degradative role [57]. Additionally, CCL3, CCL4, and CCL5 upregulated the secretion of MMP-9 by lymphocytes in vitro [58], and CCL2 increased the secretion of MMP-12 from macrophages in vitro [59], suggesting a role for these CC-chemokines in ECM degradation.

Chemokines are also involved in promoting the regression of neovessels, formed in the previous phases. Whilst this process is not fully understood, it is thought to be due to the expression of two angiostatic chemokines, CXCL10 and CXCL11. These chemokines bind to their receptor CXCR3, which is the same anti-angiogenic receptor that binds to CXCL4 in the early hemostasis phase. Binding of CXCL10 to CXCR3 prevents VEGF-induced endothelial cell tubulogenesis [20]. Similarly, activation of μ-calpain by CXCL10 cleaves β3 integrin, leading to the dissociation of endothelial cells and death [60]. Furthermore, knockout of the CXCR3 receptor in mice was found to produce wounds with a weakened healed dermis caused by insufficient remodeling and reorganization of collagen in the wound [8].

## 3. Chemokines in Complex Wounds

Wounds are not only simple open cuts or lacerations but also occur in several other forms and by various methods, including surgical incisions, combat, burns, and skin grafts. The type of wound and environment can also determine the presence of chemokines and rate of wound healing (Table 2).

### 3.1. Combat Wounds

One type of complex wound is the combat wound or blast-related wound. Wounds such as these are characterized by large injury zones affecting soft tissue, bone, and muscle and largely result in amputation. The healing of combat wounds is highly dependent on the regulation of the inflammatory response, whereby prolonged inflammation promotes prolonged wound healing times. Combat wounds are also prone to bacterial infection, which may lengthen the healing process even further. Wounds which have been sutured closed may also experience wound rupture or dehiscence. Studies have discovered the presence of several chemokines which may play a role in the prolongation of combat wound healing [61,62,63].

Patients with combat wounds which resulted in amputation were found to have increased serum levels of the pro-inflammatory chemokines CXCL8, CXCL9, and CCL5. Similarly, wound effluent from these patients had increased levels of CXCL8, CCL1, CCL2, CCL3, and CCL4 [61]. For non-amputated combat wounds, continuous maintenance and care is required to promote wound healing. This involves daily cleaning or debridement of the wound to prevent or reduce bacterial infection. During debridement, serum levels of pro-inflammatory chemokines CXCL8 and CCL3 were found to be elevated in the first and third debridement at approximately 2 and 4 days after surgical procedures in dehisced combat wounds [62]. However, the angiostatic CXCL10 was seen to be reduced in dehisced wound effluent [62]. This contrast in chemokine activity may point to a dysregulated or mixed inflammatory response. Additionally, RNA levels of CCL2 and CCL3 are increased in dehisced wounds, while CXCL10 and CCL5 are reduced, further indicating an imbalanced chemokine response in dehisced combat wounds [62]. Interestingly, combat amputation wounds were found to have decreased CXCL10 and increased CCL5 levels in the serum, whilst CCL2 and CXCL9 were decreased in the wound effluent [61].

Other than dehiscence, bacterial infection also significantly affects combat wounds. Systemic analysis of chemokines in combat wounds infected with <10^5^ colony forming units (CFU) of bacteria were found to have increased serum levels of CXCL10, CXCL8, and CCL3 compared to combat wounds without bacterial infection [63]. Similarly, local wound effluent produced elevated CXCL8 and CCL3 in bacterial combat wounds compared to nonbacterial combat wound [63]. Consistent with this, patients with amputations resulting from combat wounds showed increased serum levels of CCL3 and CCL4 as well as increased CXCL8, CCL3, and CCL4 in the wound effluent due to bacterial infection [61].

Overall, combat wounds resulting in amputation or without amputation are marked by an increased pro-inflammatory chemokine state. This is also true for wounds that experience dehiscence. Additionally, dehisced wounds have low angiostatic CXCL10 and CCL5, pointing to an irregular chemokine response. The increased pro-inflammatory chemokine state may also be due to bacterial infections which can occur from foreign debris in combat wounds. Thus, it is essential to properly clean and care for combat wounds and reduce the inflammatory response to allow for better healing of the wounds.

### 3.2. Burns

The wound healing phase in response to burn injury is similar to that of a normal wound, consisting of inflammation, proliferation, and remodeling. However, in burns, there is a higher degree of inflammation, resulting in increased capillary permeability, persistent vasodilation, and edema [64]. Cellularly, neutrophils and macrophages are responsible for removal of necrotic debris, removal of toxins, and prevention of infection of the burn [64].

Chemokines are essential to orchestrate this inflammatory response. In mice, serum CCL2, CXCL1, CCL3, and CCL11 are found to be significantly higher at day 1 post-burn injury compared to unwounded controls [65]. This reflects a significant increase in monocytes at day 3 post-burn injury, suggesting an early acute macrophage inflammatory response [65]. Interestingly, neutrophils are found to be strikingly increased in burn wounds at day 7, which may be related to the elevated CXCL1 (neutrophil chemoattractant) at day 1 [65].

In biopsies of human burns, an increase of CXCL8 3 weeks after the initial injury is associated with delayed healing [66]. In vitro, the elevation of CXCL8 was determined to reduce keratinocyte proliferation and produce less elongated fibroblasts, suggesting a delay in the re-epithelialization of the burn wound [66]. However, in a more recent study, CXCL8 was found to be reduced in the exudate of burn wounds at 11–21 days post initial injury, which is thought to allow for a more macrophage-heavy immune response such as occurs with an increase of CCL5, CCL18, and CCL27 [67]. Consistent with this macrophage immune response, CCL2, CCL3, and CCL7 are at peak levels between days 0–6 post-burn, allowing for the increased recruitment of macrophages [68]. In contrast, CXCL10 (involved in remodeling) peaks 30+ days post-burn when the macrophage presence is reduced [68]. Furthermore, if sepsis occurs in burn wounds, CCL2 is at peak levels early after the burn injury and declines over time, whilst CXCL10 is found to peak at days 7–14, with slight reduction by 30+ days post-burn [68].

Given the increased inflammatory state caused by the burn, the wound healing process is prolonged through the presence of excess pro-inflammatory chemokines. This leads to longer wound healing times with remodeling peaking later compared to a normal wound.

### 3.3. Skin Grafts

Skin grafts may be required after injuries such as burns, large open wounds, bed sores, skin infections, or skin cancer surgery. There are two main types of skin grafts: split thickness and full thickness grafts. In a split thickness graft, the epidermis and some of the dermis are replaced, and this type of graft is usually used to cover larger areas. However, in a full thickness graft, the whole epidermis and dermis is replaced. These grafts tend to cover smaller areas and closely match the recipient skin as they cover visible parts of the body, for example, the face or arms.

The immunological response to skin grafts is controlled by the use of immunosuppressants to prevent the rejection of the graft. This is particularly pertinent in an allograft, when the donor and recipient are not the same person. Following a skin graft, there is a high infiltration of neutrophils and macrophages. As new connections are established, dendritic cells from the donor tissue migrate through lymphatic vessels to the lymph nodes [69]. This causes the activation of T cells, which in turn activates the adaptive immune system, leading to skin graft rejection [69]. T cells express several chemokine receptors including CCR5 and CXCR3 on Th1 cells and CCR3, CCR4, and CCR8 on Th2 cells [70], which may help to recruit T cells to the graft site. Studies in mice reveal that allograft rejection was associated with peak expression of CCL2, CCL3, CCL4, and CXCL1 at 3 days post-transplant, however, CCL5 and CXCL10 did not peak until day 9 [71], which is consistent with the inflammatory cell content at the skin graft site. Furthermore, CXCL9 and CXCL10 were found to be expressed earlier and at higher concentrations in xenografts compared to allografts [72]. Interestingly, treatment with CXCL9 or CXCL10 antiserum increased xenograft survival in rats by 2 and 4 days, respectively [72]. Additionally, in studies where SCID mice were grafted with human skin and intraperitoneally injected with human T cells, CCL2 and CCL3 were found to play a significant role in the recruitment of human T cells directly to the grafted skin [73].

## 4. Chemokines in Chronic Non-Healing Wounds

Non-healing or chronic wounds occur when normal healing is impaired due to either a metabolic disease such as diabetes or through age-related decline in repair and regeneration. These wounds are characterized by prolonged inflammation and inadequate or weak wound closure. With an increasing ageing population and the elevated incidence of diabetes worldwide, there is a significant population that develops foot ulcers, resulting in a significant health impact to the individual and that may lead to lower-limb amputation, as very few treatments have proven therapeutic efficacy. Chronic wounds typically have an increased chemokine milieu.

### 4.1. Diabetic Wounds

Diabetes mellitus is a metabolic disease characterized by high blood glucose levels, sustained inflammation, and endothelial dysfunction. These characteristics contribute to a prolonged wound healing process. When the wound does eventually heal, the wound area is weak and prone to reinjury [74]. Several key chemokines have been studied in diabetic wounds, including CCL2, CXCL2, and CXCL12. These chemokines have been targeted for their role in sustained inflammation, formation of granulation tissue, and effects on re-epithelialization.

In diabetic mice, CCL2 expression was lower at the 24 h time point post-wounding compared to controls. However, CCL2 and CXCL2 expression was sustained throughout for an extended period of 13 days post-wounding [12]. This correlated with elevated levels of neutrophils and macrophages during the later stages of wound healing and increased expression of inflammatory cytokines IL-1β and TNF-α [12], which is not conducive to successful healing. Interestingly, in diabetic mice, neutrophils were localized to the newly formed epithelium, while macrophages were located around the neutrophils [12]. Both cells were found colocalized in granulation tissue. Furthermore, bone-marrow-derived macrophages displayed reduced chemotaxis to CCL2 and impaired wound healing despite expressing normal CCR2 [75], indicating an impaired sensitivity of this pathway.

In addition to macrophages and neutrophils, the recruitment of endothelial progenitor cells (EPCs) make important contributions to the wound healing process [76,77,78]. CXCL12 is the main chemokine involved in this process. In diabetic animals, low circulating EPC levels are correlated with low expression of CXCL12 up to 9 days post-wounding [79]. Consistent with this, genetically diabetic (*db/db*) mice express low CXCL12 up to 7 days post-wounding [80]. In diabetic mice, treatment with CXCL12 and oxygen therapy was found to significantly improve diabetic wound healing compared to controls [79]. Similarly, treatment with a CXCL12 expression plasmid increased CXCL12 expression and improved the wound healing time from 55 to 23 days in diabetic mice [80]. Furthermore, infusion of a CXCL12 antagonist significantly impaired wound healing, especially in the early wound healing phase, in *db/db* mice [81]. These wounds exhibited decreased angiogenesis, decreased granulation tissue, and increased IL-6 and CCL8 [81].

In a study comparing wound exudates from patients with chronic diabetic wounds (more than 180 days post-wound) or acute surgical wounds (13 days post-wound), it was found that there was a striking reduction in CCL5, CXCL10, and CXCL11 and an increase in CXCL8 in chronic diabetic wounds compared to acute wounds [82]. Whilst these chemokines are important in normal wound healing, they have not been extensively investigated in the diabetic wounds. The upregulation of CXCL8 may play a role in the sustained inflammatory response that occurs in diabetic wounds through its potent neutrophil chemotactic properties [83]. In contrast, the reduction in CCL5 may be affected by the production of excess nitric oxide which has been shown to impair healing [84] and suppress production of CCL5 [85]. Reduction of CXCL10 and CXCL11 levels in diabetic wound exudates may be evidence of unsuccessful advancement of wound healing. These chemokines are involved in the proliferation and remodeling of the wound. CXCL10 and CXCL11 are expressed by basal keratinocytes [44] and are involved in re-epithelialization of the wound during the proliferation phase [44,45]. Additionally, CXCL10 and CXCL11 are both involved in neovessel regression during the remodeling phase [8,60].

### 4.2. Ageing Wounds

The aged population suffers from non-healing wounds such as chronic venous leg ulcers, pressure ulcers, and diabetic foot ulcers. Although the elderly can heal most wounds, the wound healing process is often delayed compared to wounds in younger individuals. There is evidence showing that there are age-related alterations in angiogenesis [86], keratinocyte proliferation [87], delayed synthesis of new ECM [88], reduced macrophage function [89], reduced neutrophil infiltration [90], and reduction in VEGF [91], bFGF [91], and TGF-β [92,93].

In aged wounds, following the initial wounding event, there is a markedly reduced inflammatory infiltrate compared to young wounds. In wounds from young mice, neutrophils are found to peak at day 3, however older mice do not experience this same peak despite the significant increase of CXCL1 at day 1 in older mice compared to younger mice [90]. Similarly, macrophages experienced a peak at day 3 in the wounds of younger mice, whilst older mice do not experience this peak despite an increase of CCL2 and CXCL2 at day 1 [90]. Together, this may indicate an impairment in the chemotactic function of these chemokines in aged wounds. Furthermore, aged mice were found to experience improved wound healing following subcutaneous injections of macrophages at the wound site [89]. Interestingly, in burn wounds, CCL2 expression levels are 50% lower in aged mice compared to young mice at day 1, but despite this, macrophage infiltration is comparatively similar between young and old mice [94]. Finally, the expression of CXCL12 is markedly reduced in the wounds of aged mice compared to the wounds of young mice and this correlates with a reduction in the rate of wound healing, reduced formation of granulation tissue, and reduced CD31+ neovessels in aged mice wounds [95].

Together, these studies indicate that there is a reduction in chemokine function and expression in age-related wound healing which contributes to the prolonged healing times and increased wound fragility.

## 5. The Therapeutic Targeting of Chemokines to Improve Wound Healing

Due to their extensive involvement in the wound healing process of both “normal” and complex wounds, chemokines have been considered as potential therapeutic targets to improve wound healing. Several studies have either targeted key individual chemokines and chemokine receptors or performed broad-spectrum chemokine inhibition approaches. To date, studies have reported varied levels of success. Further studies are still required to determine the best strategy for manipulating chemokines to improve wound healing.

### 5.1. Single Chemokine Targeting

There are several key chemokines which have been targeted to improve wound healing. These include CXCL8, CCL2, CCL5, and CXCL12. Studies inhibiting single CC-chemokines have shown varied effectiveness. CCL2 knockout mice exhibit delayed re-epithelialization and reduced angiogenesis early in the wound repair process, whilst CCL3 knockout mice follow a normal wound healing pattern when compared to wild-type litter mate mice [96]. Recent studies have reported that there is an improvement in healing with a single administration of CCL2 onto diabetic wounds [97]. CCR1 knockout mouse models display no alteration in wound healing [98], while intraperitoneal infusion of the CCL5 antagonist Met-CCL5 was shown to improve liver fibrosis in mice and accelerate regression of fibrosis [99].

CXCL12 and its receptor CXCR4 play an important a role in trafficking bone-marrow-derived stem cells to the wound that make a significant contribution to wound neovascularization and promote re-epithelialization of the wound [100]. Additionally, local injection of CCL21 into dermal wounds was found to enhance mesenchymal stem cell engraftment during wound healing [101]. Stem cells have been shown to differentiate into macrophages, epithelial like cells, endothelial cells, and fibrocytes, which later differentiate into myofibroblasts within the wound. Additionally, wound healing is impaired in diabetics, as hyperglycemia inhibits the expression of CXCL12 from macrophages, thereby preventing the recruitment of EPCs to the wound [102]. Similarly, topical application of the CXCR4 antagonist AMD31000 improves wound healing in diabetic mice [103]. A recent study that delivered CXCL12 topically via lactobacilli vector was shown to accelerate wound healing in mice [104]. This study demonstrated that the return of pH balance in the wound caused by lactic acid from the lactobacilli vector prevents/reduces the proteolytic environment of the wound, allowing an enhanced presence of CXCL12 in the wound before it is broken down.

### 5.2. Broad Spectrum Chemokine Targeting

Several studies that have focused on targeting a single chemokine have been ineffective. Due to some redundancy in the chemokine system, a broad-spectrum approach to chemokine inhibition may have the potential to be more effective for the improvement of wound healing. Two broad-spectrum chemokine inhibitors have thus far been tested in wound healing models. There is the CC-chemokine class inhibitor “35K” and NR58-3.14, which inhibits a number of both CC and CXC chemokines.

35K is produced by the Vaccinia virus and uniquely inhibits the entire CC-chemokine class [105]. Topical application of 35K protein to subcutaneous wounds placed on the sub flanks of mice was found to enhance wound closure and neovascularization early post-wounding [106]. 35K also increased TGF-β, a pro-angiogenic and pro-repair cytokine, and the expression of the inflammatory transcription factor NF-κB. During the later remodeling phase of wound healing, 35K reduced the deposition of collagen, suggesting a reduction in scar formation. Taken together, broad-spectrum CC-chemokine inhibition with 35K may therefore present as a therapeutic strategy to enhance the healing of chronic wounds and reduce scar formation.

Another broad-spectrum chemokine inhibitor, NR58-3.14.3, has also been tested as a wound healing agent. NR58-3.14.3 inhibits both CC and CXC chemokines including CCL2, CCL3, CCL5, CXCL8, and CXCL12, all found to play a role in healing [107]. In a mouse model of intraperitoneal surgery-induced intraperitoneal adhesions, which affect the wound healing of the peritoneum, NR58-3.14.3 prevented intraperitoneal adhesion formation post-surgery and suppressed CD45^+^ inflammatory cell number [108].

## 6. Conclusions

In conclusion, chemokines play key roles at each stage of the wound healing process to help coordinate the interaction of cells within the wound. Whilst chemokines do play an important beneficial role early post-wounding, a prolonged inflammatory response that is exacerbated by chemokines can lead to the formation of chronic non-healing wounds. This suggests that approaches to chemokine inhibition in which the stage of wound healing is considered may present as a possible therapeutic strategy to improve wound healing.

## Figures and Tables

**Table 1:** Chemokines in different stages of wound healing.

Hemostasis			Inflammation			Proliferation			Remodelling		
CCL2	+	[3]	CCL1	+	[4,5]	CCL2	+++	[4,6,7]	CXCL10	unknown	[8]
CCL3	+	[3]	CCL2	+++	[4,5]	CCL3	+++	[4,6,7]	CXCL11	unknown	[8]
CCL5	+	[3]	CCL3	++	[4,5]	CXCL1	+	[9]			
CXCL1	+	[3]	CCL4	+	[4]	CXCL2	+	[9]			
CXCL4	+++	[10,11]	CCL5	+++	[4,5]	CXCL3	+	[9]			
CXCL5	+	[3]	CCL7	+	[4]	CXCL5	+	[9]			
CXCL7	+	[3]	CXCL1	++	[3,5,12,13]	CXCL6	+	[9]			
CXCL8	+	[3]	CXCL2	++	[3,5,12,13]	CXCL7	+	[9]			
CXCL12	+	[3]	CXCL5	+	[13]	CXCL8	+	[9]			
			CXCL7	+	[13]	CXCL10	+	[7]			
			CXCL8	++	[3,4,5,12,13]	CXCL11	+	[7]			
			CXCL12	+	[13]	CXCL12	+	[14,15]			

+: denotes minor increase in relative chemokine expression; ++: denotes moderate increase in relative chemokine expression; +++: denotes large increase in relative chemokine expression; CCL: CC-chemokines ligand; CXCL: CXC-chemokine ligand.

**Table 2:** Relative chemokine expression in different wound types.

	Combat Wounds			Burn Wounds			Skin Grafts			Diabetic Wounds			Aged Wounds		
**Wound tissue**	CCL2	+ (D)	[62]	CXCL8	++	[66]	CCL2	+ (M)	[71,73]	CCL2	++ (M)	[12]	CXCL1	− (M)	[90]
	CCL3	+ (D)	[62]				CCL3	+ (M)	[71,73]	CXCL2	++ (M)	[12]	CXCL12	− (M)	[95]
	CCL5	− (D)	[62]				CCL4	+ (M)	[71]	CXCL12	− (M)	[79,80]			
	CXCL10	− (D)	[62]				CCL5	+ (M)	[71]						
							CXCL1	+ (M)	[71]						
							CXCL9	+ (M)	[72]						
							CXCL10	+ (M)	[71,72]						
**Wound effluent**	CCL2	+, − (D)	[61]	CXCL8	+++	[67]				CCL5	−	[82]			
	CCL3	++, + (B)	[61,63]							CXCL8	+	[82]			
	CCL4	++, + (B)	[61]							CXCL10	−	[82]			
	CCL11	+	[61]							CXCL11	−	[82]			
	CXCL10	− (D)	[62]												
	CXCL8	++, + (B)	[61,63]												
	CXCL9	− (D)	[61]												
**Serum**	CCL3	++ (D), + (B)	[61,62,63]	CCL2	+ (M)	[65,68]									
	CCL4	++ (D), + (B)	[61,62]	CCL3	+ (M)	[65,68]									
	CCL5	++, ++ (D)	[61]	CCL7	++	[68]									
	CXCL8	++, + (B)	[61,62,63]	CCL11	+ (M)	[65]									
	CXCL9	++, ++ (D)	[61]	CXCL1	++ (M)	[65]									
	CXCL10	++ (D), + (B)	[61,63]	CXCL10	++	[68]									

All values are from human samples unless specified with: (M) to indicate mouse samples, (+) minor increase in expression, (++) moderate increase in expression; (−) decreased expression, (D) dehisced wounds, (B) bacterial infected wound.

## Abbreviations

BAOEC	Bovine aortic endothelial cell
bFGF	Basic fibroblast growth factor
ECM	Extra cellular matrix
EPC	Endothelial progenitor cells
HMEC-1	Human microvascular endothelial cell
HUVEC	Human umbilical vein endothelial cell
IFN-γ	Interferon-γ
NF-κB	Nuclear factor κB
PDGF	Platelet derived growth factor
TGF-β	Transforming growth factor β
TNF-α	Tumor necrosis factor α
VEGF	Vascular endothelial growth factor
